# Modeled Carbon Footprint of Change of Sterile Gloves and Instruments for Abdominal Wound Closure

**DOI:** 10.1001/jamanetworkopen.2025.25355

**Published:** 2025-08-06

**Authors:** Virginia Ledda, Adesoji Ademuyiwa, Adewale Adisa, Aneel Bhangu, Dhruv Ghosh, Parvez David Haque, J. C. Allen Ingabire, Faustin Ntirenganya, Maria Picciochi, Atul Suroy, Robert Lillywhite, Dmitri Nepogodiev

**Affiliations:** 1 NIHR Global Health Research Unit on Global Surgery, University of Birmingham, United Kingdom; 2 Nigeria Hub, NIHR Global Health Research Unit on Global Surgery, University of Lagos, Lagos, Nigeria; 3 India Hub, NIHR Global Health Research Unit on Global Surgery, Christian Medical College and Hospital Ludhiana, Punjab, India; 4 Rwanda Hub, NIHR Global Health Research Unit on Global Surgery, University of Rwanda, Kigali, Rwanda; 5 School of Life Sciences, University of Warwick, United Kingdom

## Abstract

**Importance:**

The Cheetah randomized trial demonstrated that changing sterile gloves and instruments before wound closure reduces surgical site infections (SSI) in abdominal surgery. However, its environmental impact remains unclear.

**Objectives:**

To estimate the global carbon footprint associated with changing sterile gloves and instruments before closure abdominal wound.

**Design, Setting, and Participants:**

This decision analytic model compared the carbon footprint of a glove and instrument change intervention against a control (no glove and instrument change). Model parameters were sourced from a large cluster randomized trial conducted in 7 low- and middle-income countries (LMICs) between June 2020 and March 2022, as well as data from stakeholder engagement and existing research. Boundaries included the trial intervention and in-hospital resources used to manage SSI. The analysis was stratified by wound contamination status (clean-contaminated, contaminated-dirty) and country-income classification.

**Main Outcome and Measures:**

Average per-patient wound-specific carbon footprint, calculated as the sum of the carbon footprint of glove and instrument change and SSI. Sensitivity analyses were based on the lowest and highest possible values for key model parameters: intervention effectiveness, intervention carbon footprint, and SSI carbon footprint. The best-case analysis was based on highest possible intervention effectiveness, lowest possible intervention carbon footprint, highest possible SSI carbon footprint. The worst-case analysis was based on lowest intervention effectiveness, highest intervention carbon footprint, and lowest SSI carbon footprint.

**Results:**

In LMICs, the difference in carbon footprints between the intervention and control groups was 10.97 kg CO_2_ equivalents (kgCO_2_e) (scenario range, −2.53 to 33.50 kgCO_2_e) for clean-contaminated and 22.60 kgCO_2_e (scenario range, −1.62 to 61.17 kgCO_2_e) for contaminated-dirty surgeries. In high-income countries, differences were 4.14 kgCO_2_e (scenario range, −3.38 to 17.95 kgCO_2_e) and 10.48 kgCO_2_e (scenario range, −3.06 to 37.62 kgCO_2_e), respectively. Country-level modeling found the intervention to be consistently associated with a lower wound-specific carbon footprint across all countries.

**Conclusions and Relevance:**

In this decision analytic model, sterile glove and instrument change before wound closure was associated with a reduced wound-specific carbon footprint across all country income settings. Alongside clinical and economic benefits, this intervention may support more sustainable surgical care; national associations and governments should consider its adoption to improve outcomes for both patients and the planet.

## Key Points

**Question:**

What is the global carbon footprint of changing sterile gloves and instruments before closure of clean-contaminated and contaminated-dirty abdominal wounds?

**Findings:**

This decision analytic model showed that, in the base case scenario in lower- and middle-income countries, changing gloves and instruments was associated with a reduction in the wound-specific carbon footprint of 10.97 kg CO_2_ equivalents (kgCO_2_e) in clean-contaminated surgeries and of 22.60 kgCO_2_e in contaminated-dirty surgeries. In high-income countries in the best-case scenario, reductions were 4.14 kgCO_2_e and 10.48 kgCO_2_e, respectively.

**Meaning:**

This model demonstrates an overall net reduction in carbon emissions with the change of sterile gloves and instruments, supporting its adoption in surgical practice.

## Teaser

This decision analytical model study evaluates the association of changing sterilized gloves and instruments in surgery with changes in wound care–specific global carbon footprint.

## Introduction

Health care contributes 4.6% to global greenhouse gas emissions, and operating theaters contribute at least 25% of the hospital carbon footprint.^1,2^ Over two-thirds of the carbon footprint of surgical consumables are due to the production and disposal of single-use products.^3^ The World Health Organization (WHO) 26th Climate Change Conference (COP26) Health Programme oversees the process of health care decarbonization, with 26 countries committed to delivering sustainable, low-carbon health systems.^4^ Twenty-two of these have set specific time limits to reach net zero targets, including the National Health System (NHS) in the UK, which aims to reach net zero by 2045.^5,6^ These targets will not be met unless the carbon footprint associated with single-use surgical consumables is addressed.^7^

Surgical site infections (SSI) are the commonest postoperative complication, disproportionally affecting patients in low- and middle-income countries (LMIC).^8^ Patients with SSIs are more likely to undergo reintervention and have a 3 times longer length of stay in hospital compared with those without SSI.^8^ While many studies have tested interventions to reduce postoperative SSI and evaluated their cost-effectiveness, very few have assessed their carbon impact.^9,10,11,12,13^ However, it is important to consider this since there is evidence that some SSI interventions may increase overall carbon footprint.^13^

The Cheetah cluster randomized trial found that changing sterile gloves and instruments prior to wound closure significantly reduced SSI following abdominal surgery compared with not changing gloves and instruments.^14^ However, there are remaining concerns that the increased use of resources, including single-use gloves and sterilization processes, may be causing an overall environmental harm. The aim of this study was estimate the global carbon footprint associated with changing sterile gloves and instruments before closure of abdominal wounds.

## Methods

### Cheetah Trial Design and Outcomes

The Cheetah trial was a multicenter, cluster randomized trial conducted in 7 LMICs (Benin, Ghana, India, Mexico, Nigeria, Rwanda, and South Africa) between June 24, 2020, and March 31, 2022.^14^ The trial aimed to determine whether changing gloves for all scrubbed staff and changing instruments before fascial closure of abdominal wounds could reduce SSI. The intervention required the use of sterile surgical gloves and 3 sterile instruments routinely needed for abdominal wound closure (needle holder, forceps, and scissors). Hospital clusters were randomized into either the intervention group (change of gloves and instruments) or the control group (routine hospital practice). Patients were eligible for inclusion if they were undergoing elective or emergency abdominal surgery with a clean-contaminated, contaminated, or dirty wound measuring 5 cm or greater. The primary outcome was the SSI within 30 days after surgery.^14^ The trial found that the rate of SSI was 18.9% (1280 out of 6768) in the routine practice group and 16.1% (931 out of 5789) in the intervention group, demonstrating that a change of gloves and instruments before the closure of abdominal wound reduced the risk of SSI (with an adjusted risk ratio of 0.87; 95% CI, 0.79-0.95; *P* = 003).^14^

Additionally, a decision analytic model was built to estimate the average costs and financial outcomes of the change in gloves and instruments. Across all assumed cost-effectiveness thresholds ($0 to $14 000), the intervention consistently showed a greater likelihood of being cost-effective than current practice, suggesting this is a cost-effective option.^15^

The reporting of this study was informed by the Consolidated Health Economic Evaluation Reporting Standards (CHEERS) reporting guideline. The NHS Health Research Authority decision tool was also utilized, which indicated that research ethics committee review was not required. We did not use generative AI at any stage in this paper.^17^

### Model Boundaries

This retrospective decision-analytic model boundaries included the trial intervention and in-hospital resources used to manage SSI (Box). It was assumed that, other than the use of the intervention and treatment of SSI, all other aspects of the initial surgery and subsequent treatment would be equivalent across groups. The study also did not include transport of staff to the hospital, or consultations and treatments provided in the community (Box).

Box. **Model Boundaries and Definitions**

**Included Within Model Boundaries**

- **Carbon footprint of trial intervention**
  - Carbon footprint of glove change: GHG emissions generated by change of gloves for scrubbed staff (kgCO_2_e)
  - Carbon footprint of instrument change: GHG emissions generated by change of sterile instruments (needle holder, forceps, scissors) (kgCO_2_e)
- **Carbon footprint of SSI (in-hospital resources)**
  - GHG emissions generated by resources used to manage SSIs within hospital confines (kgCO_2_e)

**Excluded From Model (Assumed Equal Across Groups)**

- **Carbon footprint of index operation**
  - GHG emissions generated during the patient’s index operation
- **Carbon footprint of non-SSI complication:**
  - GHG emissions generated with the management of non-SSI complications

**Excluded From Model (Outside Model Boundaries)**

- **Carbon footprint of SSI (out of hospital resources)**
  - GHG emissions generated by resources used to manage SSIs outside hospital confines

Abbreviations: GHG, greenhouse gas; kgCO_2_e, kg CO_2_ equivalents; SSI, surgical site infections.

### Wound-Specific Carbon Footprint

An average per-patient wound-specific carbon footprint (WSCF) was calculated as the sum of the carbon footprint of sterile glove (CF_GC_) and instrument change (CF_IC_) and SSI (CF_SSI_). This was calculated for both the intervention (sterile glove and instrument change) and control group (no glove and instrument change). The following equation was used to calculate the wound-specific carbon footprint:

*WSCF = CF_GC_ + CF_IC_ + CF_SSI_ × R_SSI_*.

Within this equation:

*CF_GC_* = CF_PG_
* × N_SC_ × R_GC_.*

CF_GC_ is the carbon footprint of glove change. This includes the carbon footprint of a single pair of sterile gloves (CF_PG_), multiplied for the number of scrubbed staff (N_SC_) in theater and the rate of glove change (R_GC_). To calculate the carbon footprint of instrument change (CF_IC_), we used the following formula:

*CF_IC_* = *CF_SI_* × *R_IC_.*

This was estimated assuming that additional sterilization and rewrapping of a set of 3 individual instruments (CF_SI_) would be required. This is multiplied by the rate of instrument change (R_IC_). CF_SSI_ is the carbon footprint of an SSI, and R_SSI_ is the SSI rate.

### Glove and Instrument Change Carbon Footprint

The rates of sterile glove and instrument change in routine vs interventional practice was based on data captured in the Cheetah trial (eTable 1 in Supplement 1).^14^ The carbon footprints per pair of sterile gloves and per set instruments were extracted from the HealthcareLCA database (eAppendix 1 in Supplement 1). This is an open access living repository of studies of the environmental impact of health care–related resources and services.^16^

### SSI Carbon Footprint

**Figure 1.**
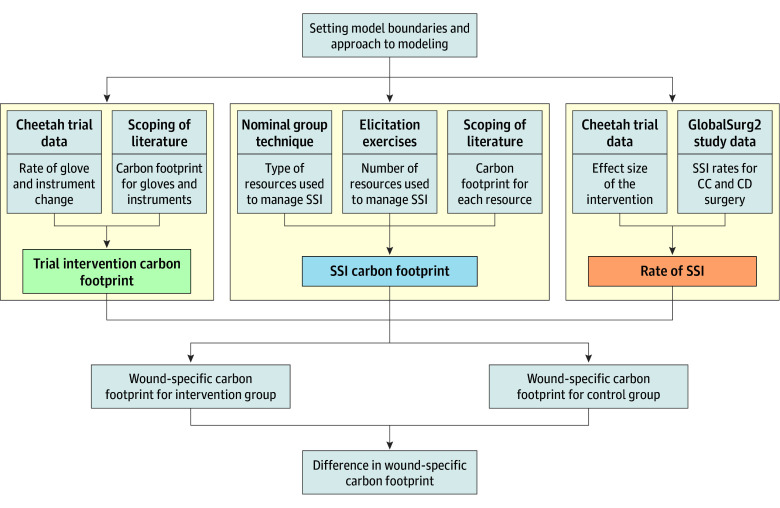
Study Flowchart and Data Sources CC indicates clean-contaminated; CD, contaminated-dirty; SSI, surgical site infections.

The data used to populate the equation above were extracted from different sources (Figure 1; eTable 2 in Supplement 1). To estimate the carbon footprint of an SSI, we first defined the resources used to manage SSI, within the predefined model boundaries. Next, for each resource, we determined the number of units used to treat SSI, and the carbon footprint per unit.

A nominal group technique (NGT) was utilized to reach consensus on the resources used to manage SSI, to be included in the model. A preliminary longlist of resources was created after scoping the existing literature, with details of the search listed in eAppendix 1 in Supplement 1. This was then finalized through discussion among the authors. NGT participants voted on the appropriateness of including each resource on the longlist in the model and had an opportunity to propose additional resources to be considered. The selection of resources to be included in the final model was finalized through group discussion.

Elicitation exercises were undertaken with experts in SSI management to determine the number of units of each resource used to treat SSI. Four elicitation exercises were completed, both in-person (1 exercise) and through online webinars (3 exercises) (eFigure in Supplement 1). The participants were asked to provide estimates of the number of additional units of each resource used to manage an SSI above and beyond the resources that would normally be used in a postoperative patient without SSI. Central estimates were based on the average across all respondents. In addition, lower and upper bound estimates were based on the interquartile range from the elicitation. Final results were stratified by respondent country income group (HIC, LMICs). The NGT meeting and elicitation exercises consisted in stakeholder engagement, as fully anonymized expert opinions from our network of research collaborators and coauthors were collected, with no patients involved.

The HealthcareLCA database was used to estimate the carbon footprint per unit of each resource used to manage SSI.^16^ Details of this search are presented in eAppendix 1 in Supplement 1. If the carbon footprint of a resource could not be found in the HealthcareLCA database, a scoping search was conducted to identify alternative data sources (eAppendix 1 in Supplement 1). If there were multiple carbon footprint values available for a particular resource, the central estimate was based on an average and lower and upper bound estimates were based on the lowest available and highest available value, respectively. Further details of the methodology are explored in eAppendix 2 in Supplement 1.

### SSI Rates

The baseline SSI rate was taken from the GlobalSurg 2 international, multicenter, prospective cohort study which characterized variation of SSI rates across high, low-middle, and low income countries.^8^ The study included 12 539 participants from 343 hospitals in 66 countries. The study showed that countries with low Human Development Index (HDI) had higher SSI rates compared with high-HDI countries. The study produced country-level SSI rate estimates, stratified by the degree of intra-operative contamination. We calculated pooled median SSI rates for HICs and LMICs, based on World Bank income classification (high, upper-middle, lower-middle, and low income countries), stratified by contamination grade (clean-contaminated, contaminated-dirty surgery).^8^

### Statistical Analysis

Carbon modelling was conducted in Excel version 16.98 (Microsoft). Given the nature of the modelling, which was based on using input values rather than statistical modelling, we did not apply statistical significance testing. The focus was on estimating carbon differences under defined assumptions across country and contamination scenarios.

#### Base Case and Sensitivity Analyses

In addition to the base case, we also modeled worst- and best-case scenarios for the intervention effectiveness. For the base case we used (1) the point estimate for the effectiveness of the intervention, (2) central estimates for the carbon footprint of the intervention, and (3) the central estimates for the number of additional units of resources and their associated carbon footprint. The worst-case scenario was based on (1) the upper bound of the 95% CI for the intervention effectiveness from Cheetah, (2) upper bound estimates for the intervention carbon footprint, and (3) the lower bound estimates for the SSI carbon footprint. The best-case scenario was based on (1) the lower bound of the 95% CI for the intervention effectiveness from Cheetah, (2) lower bound estimates for the intervention carbon footprint, and (3) the upper bound estimates for the SSI carbon footprint. Details of the parameters used across different scenarios are summarized in eTable 3 in Supplement 1.

#### Country-Level Analysis

A preplanned secondary analysis was undertaken to model intervention impact at country-level. This analysis was based on country-specific data from GlobalSurg-2 study for SSI rates following clean-contaminated and contaminated-dirty surgery.^8^ Country-level estimates were based on base-case assumptions with regards to SSI and intervention carbon footprints.

## Results

### NGT Meeting and Elicitation Exercises

A total of 16 participants were included in the NGT meeting (eTable 4 in Supplement 1). During voting and the following discussion, agreement was reached on the inclusion of the following resources within the model: wound swabs used to collect specimens from the wound and their analysis, wound dressing, and oral antibiotics for the treatment of SSI. The resources considered for the estimation of the carbon footprint of an SSI also included reattendance, readmission to the hospital, reoperation, and length of stay (eTable 5 in Supplement 1). Other resources were suggested for inclusion in the model, and outcomes on their inclusion are explored in eTable 6 in Supplement 1. A mix of 198 participants from HICs (97 participants [49.0%]) and LMICs (101 participants [51.0%]) was included in the elicitation exercises (162 surgeons [81.8%], 3 nurses [1.5%]; 155 university hospital referrals [78.3%]) (eTables 7 and 8 in Supplement 1). Results of the elicitation exercises are displayed in eTable 9 in Supplement 1.

### Carbon Footprint of the Intervention

The parameters used to estimate the carbon footprint of the sterile glove and instrument change are summarized in eTables 10 and 11 in Supplement 1. Overall, considering contamination across trial groups, the carbon footprint of sterile glove and instrument change in the intervention group was 2.66 kgCO_2_e (lower bound, 1.73 kgCO_2_e; upper bound, 3.71 kgCO_2_e) and in the control group was 0.02 kgCO_2_e (lower bound, 0.01 kgCO_2_e; upper bound, 0.03 kgCO_2_e).

### Carbon Footprint of SSI

The parameters used to estimate the carbon footprint of an SSI are summarized in Table 1 and eTable 11 in Supplement 1. While some resource usage estimates were consistent across income groups, such as wound swabs (median [IQR] 2 per SSI for LMICs [1-3 swabs] and HICs [1-5 swabs]) or days of antibiotics (median [IQR] 7 days to manage an SSI for LMICs [7-10 days] and HICs [3-6 days]), some notable differences were observed. Specifically, the length of stay, defined as the additional days of inpatient admission for the management of an SSI, was a median (IQR) 7 days in LMICs (5-7 days), while only 5 days in HICs (3-6 days). As the carbon footprint for a day of inpatient admission was estimated as 106 kgCO_2_e per day, this difference is reflected in the overall carbon footprint difference among country income groups. In fact, the estimated SSI carbon footprint in LMICs was 764.23 kgCO_2_e (lower bound, 164.43 kgCO_2_e; upper bound, 1197.30 kgCO_2_e) and in HICs was 553.34 kgCO_2_e (lower bound, 100.85 kgCO_2_e; upper bound, 1038.35 kgCO_2_e). The estimated baseline SSI rate was estimated as 9% for clean-contaminated and 18% for contaminated-dirty surgery in HICs, and 14% for clean-contaminated and 25% for contaminated-dirty surgery in LMICs. Country-level SSI estimates are presented in eTable 12 in Supplement 1.

**Table 1.** Parameters for Calculation of the Carbon Footprint of a Surgical Site Infection

Resources included	Carbon footprint per resource, kgCO_2_e per unit (scenario range)^a^	Resource used, median (IQR)^a^	
		LMICs	HICs
Wound swabs, No.	0.61 (0.61-0.61)	2 (1-3)	2 (1-5)
Wound dressings, No.	0.03 (0.01-0.06)	9 (5-12)	7 (5-10)
Time of antibiotic, d	0.02 (0.01-0.02)	7 (7-10)	7 (5-7)
Reoperation rate, %	147 (112-184)	2 (1-5)	2 (1-5)
Length of stay, d	106 (32-161)	7 (5-7)	5 (3-6)
Reattendance rate, %	29.3 (22-67)	6 (3-15)	10 (5-16.5)
Readmission rate, %^b^	106 (32-161)	5 (2-10)	5 (2-10)

Abbreviations: HICs, high-income countries; kg CO_2_e, kg CO_2_ equivalents; LMICs, low- and middle-income countries.

^a^ Scenario ranges represent the values used for best and worst case scenarios. For carbon footprints, the scenario range was based on lowest and highest possible values extracted from prior research. For resource usage, the scenario range was based on interquartile ranges from the elicitation exercise (See eTable 3 in Supplement 1).

^b^ Assumed average length of stay on readmission is 3 days.

### Base Case

In LMICs, for clean-contaminated surgery the wound-specific carbon footprint was 10.97 kgCO_2_e lower per patient in the intervention (93.77 kgCO_2_e) than the control group (104.74 kgCO_2_e), and in contaminated-dirty surgery, it was 22.60 kgCO_2_e lower per patient (171.57 kgCO_2_e vs 194.17 kgCO_2_e) (Table 2). In HICs, for clean-contaminated surgery the wound-specific carbon footprint was 4.14 kgCO_2_e lower per patient in the intervention (48.03 kgCO_2_e) than the control group (52.17 kgCO_2_e), and in contaminated-dirty surgery, it was 10.48 kgCO_2_e lower per patient (90.51 kgCO_2_e vs 100.99 kgCO_2_e) (Table 2).

**Table 2.** Wound-Specific Carbon Footprints for Intervention and Control Groups, Stratified by Contamination Grades and Income Groups

	Base scenario, kgCO_2_e			Best-case scenario, kgCO_2_e			Worst-case scenario, kgCO_2_e		
	Control group	Intervention group	Difference^a^	Control group	Intervention group	Difference^a^	Control group	Intervention group	Difference^a^
**LMICs**									
Clean-contaminated	104.74	93.77	10.97	167.72	134.22	33.50	23.05	25.58	−2.53
Contaminated-dirty	194.17	171.57	22.60	299.49	238.32	61.17	41.13	42.76	−1.62
**HICs**									
Clean-contaminated	52.17	48.03	4.14	93.68	75.73	17.95	6.22	9.60	−3.38
Contaminated-dirty	100.99	90.51	10.48	187.34	149.72	37.62	12.42	15.48	−3.06

Abbreviations: HICs, high income countries; kg CO_2_e, kg CO_2_ equivalents; LMICs, low- and middle-income countries.

^a^ Positive values indicate a reduction in wound-specific carbon footprint with the use of the intervention, while negative indicate an increase in wound-specific carbon footprint.

### Sensitivity Analyses

In LMICs, the best-case scenario estimated a wound-specific carbon footprint that was 33.50 kgCO_2_e lower per patient (134.22 kgCO_2_e vs 167.72 kgCO_2_e) in clean-contaminated surgery and 61.17 kgCO_2_e lower (238.32 kgCO_2_e vs 299.49 kgCO_2_e) in contaminated-dirty surgery in the intervention group compared with the control. In the worst-case scenario the model estimated a small increase of 2.53 kgCO_2_e (25.58 kgCO_2_e vs 23.05 kgCO_2_e) in clean-contaminated and 1.62 kgCO_2_e (42.76 kgCO_2_e vs 41.13 kgCO_2_e) in contaminated-dirty surgery in the intervention group (Table 2).

In HICs, in the best-case scenario, the wound-specific carbon footprint for clean-contaminated surgery was estimated as 17.95 kgCO_2_e lower per patient in the intervention than the control group (75.73 kgCO_2_e vs 93.68 kgCO_2_e), and 37.62 kgCO_2_e lower per patient (149.72 kgCO_2_e vs 187.34 kgCO_2_e) in contaminated-dirty surgery. However, in the worst-case scenario the model estimated a small increase in the wound-specific carbon footprint of 3.38 kgCO_2_e (9.60 kgCO_2_e vs 6.22 kgCO_2_e) in clean-contaminated and 3.06 kgCO_2_e (15.48 kgCO_2_e vs 12.42 kgCO_2_e) in contaminated-dirty surgery in the intervention group compared with the control (Table 2).

### Country-Level Analyses

**Figure 2.**
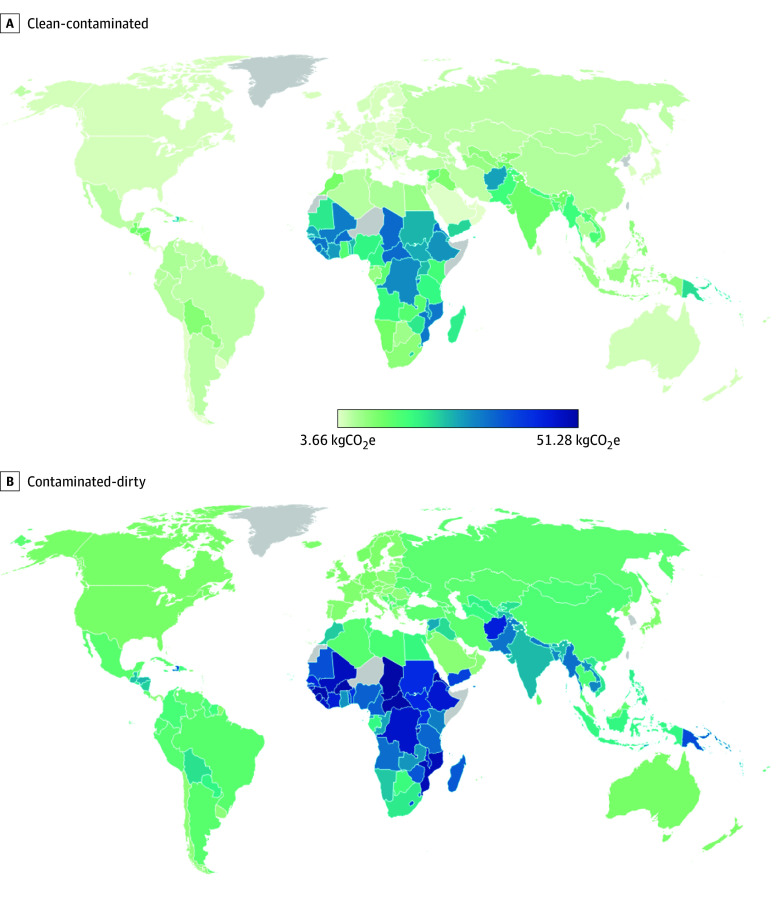
Country-Specific Net Carbon Reduction With the Sterile Glove and Instrument Change in Clean-Contaminated and Contaminated-Dirty Surgery Country-level estimates were based on base-case assumptions with regards to surgical site infections and intervention carbon footprints.

In the country-level analyses, the wound-specific carbon footprint was found to be lower in the intervention than the control group across all countries for both clean-contaminated and the contaminated-dirty surgery (Figure 2). The carbon footprint reductions associated with the trial intervention varied widely across countries, ranging from 3.66 kgCO_2_e to 34.15 kgCO_2_e for clean-contaminated surgery, and from 9.68 kgCO_2_e to 51.28 kgCO_2_e in contaminated-dirty surgery (Figure 2). Modeled reductions were greatest in low-income countries, which was attributable to higher baseline SSI rates and greater carbon footprints associated with managing SSI, as reported previously (eTable 12 in Supplement 1).

## Discussion

This retrospective decision-analytic model demonstrates that implementing sterile glove and instrument change prior to the closure of abdominal wounds results in an overall reduction of the wound-specific carbon footprint. While this intervention was associated with a reduction in both HICs and LMICs, the greatest benefits were in LMICs and particularly in the lowest-income countries. These findings provide evidence suggesting a net benefit to using additional sterile gloves and instruments at the time of wound closure. When considered alongside previously identified clinical and cost advantages, the results strengthen the case for implementing this intervention in clinical practice.^14,15^

There is growing interest in evaluating the environmental impact of surgical interventions alongside their clinical outcomes. For example, a systematic review and meta-analysis of reusable vs disposable surgical headwear found that reusable headwear did not increase SSI rates and had a significantly lower carbon footprint.^11^ Another systematic review, assessing the use of triclosan coated sutures across 31 randomized controlled trials across LMICs and HICs, found a reduced incidence of SSIs associated with their use, and suggested potential environmental benefits.^13^ That study also provided an estimate of the carbon footprint of an SSI, although the estimate was based on low-quality clinical data and UK reference data.^13,18^ In addition, 2 UK based studies set in tertiary centers, a single-center pilot cohort study, and a 2-center observational study used digital postoperative follow-up data to estimate the carbon footprint of an SSI, based on the resource use of patients diagnosed with an infection (8 and 9 patients, respectively).^19,20^

With health care systems striving to reach net-zero targets, it is imperative that future randomized trials assess not only clinical and cost, but also carbon, outcomes within their main phase.^21^ This would enable the generation of pragmatic, high-quality evidence, to inform clinical guidelines and practice. To improve future modeling, studies are needed to address the significant gaps in our understanding of carbon footprints in LMIC settings, particularly taking in to account differences in supply chains, resource usage, and waste processing.

### Strengths and Limitations

Our study has both strengths and limitations. A key strength is that it is the first country income–stratified modeling study to assess the carbon impact of a perioperative intervention. By producing separate estimates for clean-contaminated and contaminated-dirty surgery, it enables a more personalized approach to perioperative management. Additionally, it has produced up-to-date estimates of SSI carbon footprints for both LMICs and HICs, that can inform future modeling studies evaluating other SSI prevention measures. This methodological approach is reproducible and may allow for rapid estimation of carbon impact of future trial intervention.

Limitations of the study include that it was not possible to reflect the likely variation in country-level supply chains and carbon footprints. The carbon footprints used were taken from published studies that have applied inconsistent approaches to life cycle assessment and model boundaries. The resources used to manage SSIs to be included in the model were selected through an NGT meeting, which predominantly included participants from LMICs, and this may not accurately reflect resource usage in higher-income settings. Oral antibiotics were used as a surrogate of antimicrobial treatment for SSIs, which does not reflect the wide variation of types and routes of antibiotics administered globally for SSIs. Resource usage was based on an elicitation exercise rather than patient-level data and limited to in-hospital model boundaries, potentially underestimating the true carbon footprint of SSI and therefore the carbon impact of the intervention.

## Conclusions

In this decision-analytic model, sterile glove and instruments change before wound closure was associated with a reduced wound-specific carbon footprint across all country income settings. At a time when the effects of climate change are becoming more evident and given the significant contribution of surgery to the carbon footprint of health care, estimating the carbon footprint of trial interventions has become an important step necessary to translate evidence into practice. This study demonstrates a practical approach to integrating carbon modeling into the evaluation of clinical trial interventions, moving toward more comprehensive assessments that consider environmental alongside clinical and economic outcomes. Our findings suggest that the sterile glove and instrument change not only improves patient outcomes and is cost-effective, but it is also likely to lower carbon emissions. Incorporating sterile glove and instrument changes into routine clinical practice may offer benefits for both patient outcomes and environmental sustainability and could be considered for broader implementation across hospitals.

## Notes

Supplement 1. **eAppendix 1.** Literature Scoping Summaries **eAppendix 2.** Methodology of Data Sourcing for Model Parameters and Results **eTable 1.** Rate of Glove and Instrument Change in the Cheetah Trial **eTable 2.** Data Sources for Model Parameters **eTable 3.** Parameters Used in Main Model and Sensitivity Analyses **eTable 4.** Country of Work of Participants to Nominal Group Technique Meeting **eTable 5.** Nominal Group Technique Meeting Voting Results **eTable 6.** Resources Suggested for Inclusion in the Model During Nominal Group Technique Meeting **eTable 7.** Country of Work of Participants to Elicitation Exercises **eTable 8.** Demographics of Participants to Elicitation Exercises **eTable 9.** Elicitation Exercises Result Indicating Average Use of Each Resource in the Management of an SSI **eTable 10.** Parameters for Calculation of Carbon Footprint of Glove and Instrument Change **eTable 11.** Studies Used to Extract the Carbon Footprint of Resources for Estimating the SSI of Glove and Instrument Changes, as Well as the SSI Carbon Footprint **eTable 12.** Country-Specific SSI Rates for Clean-Contaminated and Contaminated-Dirty Procedures Extracted From GlobalSurg Study **eFigure.** Flowchart of Votes From Elicitation Exercises Included in the Study
eReferences

Supplement 2.
Nonauthor Collaborators

Supplement 3.
Data Sharing Statement
