# Supplementary material for: Modeled Carbon Footprint of Change of Sterile Gloves and Instruments for Abdominal Wound Closure
Source: JAMA Netw Open. 2025 Aug 6;8(8):e2525355. doi: 10.1001/jamanetworkopen.2025.25355 (PMC12329605; doi:10.1001/jamanetworkopen.2025.25355)
Supplement: Supplement 1. — eAppendix 1. Literature Scoping Summaries eAppendix 2. Methodology of Data Sourcing for Model Parameters and Results eTable 1. Rate of Glove and Instrument Change in the Cheetah Trial eTable 2. Data Sources for Model Parameters eTable 3. Parameters Used in Main Model and Sensitivity Analyses eTable 4. Country of Work of Participants to Nominal Group Technique Meeting eTable 5. Nominal Group Technique Meeting Voting Results eTable 6. Resources Suggested for Inclusion in the Model During Nominal Group Technique Meeting eTable 7. Country of Work of Participants to Elicitation Exercises eTable 8. Demographics of Participants to Elicitation Exercises eTable 9. Elicitation Exercises Result Indicating Average Use of Each Resource in the Management of an SSI eTable 10. Parameters for Calculation of Carbon Footprint of Glove and Instrument Change eTable 11. Studies Used to Extract the Carbon Footprint of Resources for Estimating the SSI of Glove and Instrument Changes, as Well as the SSI Carbon Footprint eTable 12. Country-Specific SSI Rates for Clean-Contaminated and Contaminated-Dirty Procedures Extracted From GlobalSurg Study eFigure. Flowchart of Votes From Elicitation Exercises Included in the Study eReferences [file jamanetwopen-e2525355-s001.pdf]

Supplemental Online Content

Ledda V, Ademuyiwa A, Adisa A, et al; On behalf of the National Institute for Health and Care Research Global Health Research Unit on Global Surgery. Modeled carbon footprint of change of sterile gloves and instruments for abdominal wound closure. *JAMA Netw Open.* 2025;8(8):e2525355. doi:10.1001/jamanetworkopen.2025.25355

eAppendix 1. Collaborators

eAppendix 2. Literature Scoping Summaries

eAppendix 3. Methodology of Data Sourcing for Model Parameters and Results

eTable 1. Rate of Glove and Instrument Change in the Cheetah Trial

eTable 2. Data Sources for Model Parameters

eTable 3. Parameters Used in Main Model and Sensitivity Analyses

eTable 4. Country of Work of Participants to Nominal Group Technique Meeting

eTable 5. Nominal Group Technique Meeting Voting Results

eTable 6. Resources Suggested for Inclusion in the Model During Nominal Group Technique Meeting

eTable 7. Country of Work of Participants to Elicitation Exercises

eTable 8. Demographics of Participants to Elicitation Exercises

eTable 9. Elicitation Exercises Result Indicating Average Use of Each Resource in the Management of an SSI

eTable 10. Parameters for Calculation of Carbon Footprint of Glove and Instrument Change

eTable 11. Studies Used to Extract the Carbon Footprint of Resources for Estimating the SSI of Glove and Instrument Changes, as Well as the SSI Carbon Footprint

eTable 12. Country-Specific SSI Rates for Clean-Contaminated and Contaminated-Dirty Procedures Extracted From GlobalSurg Study

eFigure. Flowchart of Votes From Elicitation Exercises Included in the Study

eReferences

This supplemental material has been provided by the authors to give readers additional information about their work.

© 2025 Ledda V et al. *JAMA Network Open*.

eAppendix 1. Collaborators

Abbey-Louise Matthews, Adamu Issaka, Ahmad Hassan Jibril, Ahmed Abdisamed, AHMED ALBAGIR ALI ALTAYYEB, Ahmed Mekki, Aidan Bannon, Aikaterini Karakonstanti, Aime HIRWA, albaro jose nieto-calvache, Alejandro Gonzalez-Ojeda, Alfie J Kavalakat, Amar Odedra, Amman Malik, Andrea Nickeas, Andrew Stevenson , Andrey Litvin, Angelika Kaufmann, Anil Luther, Anis Hasnaoui, Anisa Kushairi, Anja Imsirovic, Anne Robinson, Antonio Perez-Ferrer, Antonio Ramos De-la Medina, April Camilla Roslani, Aristeidis Papadopoulos, Anu Susan George, Arun Sahni, Ashish Chaudhrie, Ashish Tirkey, Ashly Thomas, Ayesha Bibi, Bashir abobaker albakosh, Binay Kumar, Branko Bogdanic, Bruno Nardo, Bryony David, caitlin brennan, Cara Hatcher, Carolina Moreno Licea, Caroline Wilburn, Catriona Frankling, CHAMAIDI SARAKATSIANOU, CHARITAKI EVGENIA, Chinar Goyal, Chris J Smart, Christian Agbo, Christian Udu Ngwu, Christiana Osei-Dwomoh, Christopher Aboah, Claudia Castellanos, Cleo Kenington, CLOTILDE FUENTES OROZCO, Cortland Linder, CYNTHIA AYODEJI AGBONROFO, Deena Harji, Deepak Jain, Deepak Singh, Dimitrios Spinos, Djifid Morel Seto, Dmitry Adamovich, Dorothy Kufeji, Doug Bowley, Dr Abubakar Bala Muhammad, Dr Gareth Thompson, Dr Narendra Siddaiah, Dr Subham Jakhar, Dragana Zivkovic, Ebenezer Kwame Amofa, Ebere Osinachi Ugwu, Eleanor Cotton, Elisa Paoluzzi Tomada, Elizabeth Li, Elizabeth Westwood, Ella Wheeley, Emmanuel A. Nachelleh, Emmet Dorrian, Eseenam Agbeko, Ewen Harrison, fahed gareb, Fareeda Galley, Fennie Sam, Feriha Fatima Khidri, Francesco Pata, Gianluca Pellino, Gonzalo Delgado-Hernández, GUILLERMO YANOWSKY REYES, Gustavo Miguel Machain, Habeeb Hanafi, Hadijat Olaide Raji, HANA SIDDIG HAMMAD HASSAN, Haris Kuralić, Harry Wilson, Helen van Vliet, Helen Suttenwood , Hesham Abozied, Hesham Zalghana, hossam Eslam Mohammed salah , Hugh Montgomery, Humaira Hussain, Ibrahim Adel Hamdoun, Ifeanyichukwu Chinedum Ugwu, Imtiaz Wani, Iniesta aurelie, Isam Bsisu, Ismail LAWANI, Isobel seddon, JAMEEL ISMAIL AHMAD , James Glasbey, Jane Barnard, Jayan Dewantha Jayasinghe, Jennifer Kirkby , Jennifer Ip, Jessica Fleminger, Joël L. Lavanchy, John Tabiri Abebrese, Jon LACY-COLSON, Jonathan Lee, Jonathan P Evans, Kai Hui Loo, Katherina McEvoy, Kethy FAGNON, Khaled Mahmoud Ahmed Omar, Khaled Mohammed Al-Sayaghi, Konstancja Tadrak, Kriscia Vanessa Ascencio Diaz, Lanre Lamid, Laura Balance, Lofty-John Chukwuemeka Anyanwu, Lovenish Bains, Ludger Barthelmes, Luke Nicholson, Madushika Rajapakse, Margot Flint, Mario Jesús Guzmán Ruvalcaba , Mark Cheetham, Marta Wachtl, Massimiliano Veroux, Matthew Gardiner, Matthew Popplewell, Michelle Spiteri, Miguel GASAKURE, Minale Merene, moath Ahmed Abdullah almuradi, Mohamed Thaha, Mohamed Ghula, Mohammad Marar, Mohammed Sheriff, Mohammed Salele Aliyu, Montassar Ghalleb, Moses Dokurugu, Muhammad Fairuz Shah Abd Karim, muhammad farzreen bin mohd ismail, Muhammad Mudasir Khan, Navneet Kumar Chaudhary, Nick Battersby, Nida Wahid Bashir, Nnaemeka Nwafulume, Omolara Williams, panna patel, Pariza Gupta, Patrick Sharman, Paul Marriott, Paul Robinson, PETER IKPONMWOSA AGBONROFO, Peter Paal, Rachel Sam, Rahel Abebayehu Assefa, Raja Haseeb Basit Rajive Jose, Rajkumar KS, RAM PRASAD SUBEDI, Ramanpreet Kaur, rasiah bharathan, Rawoof Mohammed, Reddy Abhinaya P, Robert Parker, Robert Whitham, Rohin Mittal, romain Letartre, Romy Kenyon, Rory F Kokelaar, Ross Lathan, Ross Coomber, Ruzaimie Noor, Salih Al-Ani, Saminu Muhammad, Samson Olori, Samuel Ali Sani, Samuel Kwame Amoako Asirifi, Sanjay pandanaboyana, Sankar Balakrishnan, Setthasorn Ooi, Shilpa Sharma, Shireen Anne Nah, Shivani Aggarwal, Simon Clarke , Sonia Bhangu, Sonia Mathai, Soyombo Orsoo, Spiros Delis, Stefan Welter, Stelian Stefanita Mogoanta, Stephen Gboya Gana, Suraiya Auwal Suleiman, Taiye Taibat Ibiyeye, Tariq Alhammali, Theophilus Anyomih, Theophilus Justus Kofi Adjeso, Thida Oung, Tom Challoner, Tosin Olusoga Akinyemi, Upamanyu Nath, Uzair Khan, Virginie Pollet, Vairavan Narayanan, Vandana chaukar, Vasanthika Thuduvage, W M C Alwis, Wegene Tadesse Shenkutie, Yousuf Sabah, Zahra Hussain.

© 2025 Ledda V et al. *JAMA Network Open*.

eAppendix 2. Literature scoping summaries

1. **Search to identify carbon footprint of resources to be included in the model**

Objective: To obtain estimates of carbon footprint for each resource included in the model
Search strategy: We searched HealthcareLCA in first instance with the name of the resources included in the model such as *sterile gloves, sterile instruments, wound swabs, wound dressings*. For those not identified in the HealthcareLCA[1] we scoped PubMed, Embase and Google Scholar. The study was completed between the 30th May and the 7th June 2024.
Selection criteria: Studies were reviewed and included if they provided an estimate of carbon footprint for the resources selected.
Results: Studies selected are reported in Supplementary Table 11.

2. **Search on studies looking at resources used to manage SSI**

Objective: To identify resources used for the inpatient management of SSIs
Search strategy: We searched PubMed, Embase and Google Scholar with search term such as *surgical site infection, wound management, resources, services, complications, post-operative, inpatient management.* The search was conducted on the 15th May 2024.
Selection criteria: Studies were included if they suggested resources used in the management of SSIs.
Results: Studies were reviewed and longlist of resources compiled. The final list was agreed by the co-authors of the manuscript (Supplementary Table 5).

3. **Search on studies looking at the environmental impact of sterile glove and instrument change**

Objective: To identify studies addressing the environmental impact of changing gloves and instrument prior to closure of abdominal wound
Search strategy: We searched PubMed, Embase and Google Scholar with search terms such as *gloves, instruments, carbon footprint, GHG emissions, surgery*. No limitation was put on the search. The search was performed on the 27th December 2024.
Selection criteria: Studies were included if they offered an estimate of any type of environmental impact for glove or instrument change, or both.
Results: No studies were identified.

© 2025 Ledda V et al. *JAMA Network Open*.

4. **Search to identify studies looking at the environmental impact of SSI-reducing interventions**

Objective: To identify studies addressing the environmental impact of ani SSI-reducing intervention

Search strategy: We searched PubMed, Embase and Google Scholar with search terms such as *prevention or reduction*, *surgical site and wound infections*, and environmental sustainability factors such as *carbon footprint, emissions, eco-friendly and surgery, perioperative* terms. The term was conducted on the 27th December 2024.

Selection criteria: Studies were included if they provided an estimate of environmental impact of interventions.

Results: Six studies were identified, of which two focused on digital based detection of SSI and provided estimates of SSI carbon footprint, which were included in the discussion. A study on triclosan coated sutures and reusable hats in operating theatres were included.[2,3] A randomised controlled trial on ankle surgery was excluded as the intervention was not aimed at reducing the SSI rate.[4] An observational study on laminar flow in cardiac surgery was excluded as no difference in SSI rate across the two groups.[5]

© 2025 Ledda V et al. *JAMA Network Open*.

eAppendix 3. Methodology of data sourcing for model parameters and results

Phase 1: Resource selection

While the additional resources needed for the trial intervention were determined by the trial (use of additional pair of gloves per scrubbed staff and of sterile instruments), establishing the resources to be considered for the estimation of the carbon footprint of a surgical site infection (SSI) required several voting processes.

The Nominal Group Technique (NGT) is a technique of consensus development, which aims to gather input from participants through a structured face-to-face group meeting.[6] This technique is growingly employed in health-care research, where it has been used to prioritise topics, to reach expert consensus and to create diagnostic frameworks.[7-9] The purpose of NGT within this study, was to identify the additional resources which are commonly employed in the management of SSI within the study boundaries. This technique was chosen as through its structured approach it provides opportunities for all participants to share ideas, therefore ensuring equal participation. This aspect of the NGT has been found to be particularly relevant as the study aims to explore views from a variety of professionals (Surgical trainees, Surgical consultants, registered nurses) from different socioeconomic backgrounds across a spectrum of countries, encompassing a range of income groups. The NGT format also allows participants to receive immediate feedback, which allows for clarification of ideas and responses through the process.

An NGT exercise to reach consensus on the resources to include to estimate the carbon footprint of an SSI took place during the NIHR Global Surgery Unit annual meeting, which was help in Lagos on the 10th of September 2023.[10]

An initial list of resources discussed during the NGT meeting was selected based on scoping of the existing literature (Appendix 1) and expert consensus of the authors. During the preparation stage of the NGT meeting, the participants were selected among those regularly managing a surgical site infection, and briefed, with an overview of the Cheetah trial and the aims of the carbon model.

The participants were asked to review the current list of resources included, and to vote whether they agreed each resource should be included or not. Following the vote, if the results were not unanimous, inclusion of resources was discussed within the NGT until consensus was reached.

Phase 2: Defining the number of resources used

Once the list of resources to be included in the model to estimate the carbon footprint of an SSI was finalised, the average number of resources used in the management of a patient with SSI needed to be estimated. To do this, several elicitation processes were arranged. Through this process, data was collected based on the participant’s experience. By involving

multiple professionals from diverse socioeconomic backgrounds, data was triangulated among different participants and through different elicitation sessions.
The elicitation process involved surgical trainees, Consultant surgeons, registered nurses and any healthcare professional regularly in charge of managing patients with SSIs on the wards, across a variety of country from different income groups. This allowed to obtain more accurate data with regards to the number of resources which are regularly utilised in the management of SSIs.

Surveys were used to support the elicitation processes and to record their results. This technique was chosen as it was felt that it could support a pragmatic elicitation process, by supporting the facilitator and allowing recording of the results. The survey provided a short introduction to the topic and the aim of the process. The leader of the session was selected among those working in the country or region that the session was aimed to, to achieve a trusted facilitator-led elicitation process.

Phase 3: Carbon footprint of resources

After having obtained a list of resources used to manage SSIs and the average use of each resource for the management of a patient with SSI, the carbon footprint of each resource was obtained, to calculate the wound-specific carbon footprint.
The carbon footprint of each resource was based on existing literature and LCAs. The Healthcare LCA, launched in December 2021, is an open-access living repository containing studies focusing on the estimation of the environmental impact of healthcare-related resources and services. This contains research papers, reviews and pieces of grey literature.[1] The carbon footprint of resources previously identified through the NGT meeting, but also of the additional usage of gloves and instruments, was calculated using the estimated values identified through scoping of the HealthcareLCA database. If the carbon footprint of a resource could not be found on this repository, a wider scoping review will be conducted, using PubMed, Embase and Google Scholar.
Where more studies are identified looking at one resource, the most closely comparable study will be chosen following discussion among the authors, or an average of the values found will be used in the present study. In case no study was found to provide measurement of the carbon footprint of a specific item, studies looking at similar resources were considered.

Results

NGT meeting

All resources included received a minimum of 13 votes in agreement with their inclusion. Imaging (ultrasound scan, USS) only received 10 votes in agreement, and a discussion on

this concluded that USS is seldom used in clinical practice for the diagnosis of surgical site infections which involved the skin only, rather than any deep-seated infection. USS was therefore removed from the resources included in the model.

An opportunity was given to suggest additional resources for their inclusion. The suggested resources and the outcome on their inclusion can be explored in Supplementary Table 6.

Elicitation exercises

The different elicitation exercises took place during ad-hoc webinars and during a carbon model session at the Research for Greener Surgery Conference (Supplementary Figure 1).[11] Each ad-hoc webinar was arranged to facilitate the participation of healthcare professionals working in different regions (India, West Africa and the UK). The elicitation exercises included a standardised presentation with a brief overview of the model, its methodology and aim, and then a voting session. A total of 263 votes were collected from an equal number of participants. Details on participants' countries of origin and demographics are provided in Supplementary Tables 7 and 8.

The data collected through each elicitation exercise was analysed and stratified by income group (HIC and LMIC according to World Bank classification).

© 2025 Ledda V et al. *JAMA Network Open*.

eTable 1. Rate of glove and instrument change in the Cheetah trial

	Intervention arm	Control arm
Total number of participants	6,144	7,157
Number of procedures with glove change	6,048	55
Number of procedures with no glove change	96	7,102
**Rate of glove change**	**98.4%**	**0.8%**
Number of procedures with instrument change	6,047	13
Number of procedures with no instrument change	97	7,144
**Rate of instrument change**	**98.4%**	**0.2%**

© 2025 Ledda V et al. *JAMA Network Open*.

eTable 2. Data sources for model parameters

Model parameter	Data source
Gloves and instrument carbon footprint	
Rate of glove change (stratified by intervention / control trial arm)	Cheetah trial data[12]
Rate of instrument change (stratified by intervention / control trial arm)	Cheetah trial data[12]
Carbon footprint for gloves and instruments	Published literature
Surgical site infection carbon footprint	
Type of resources used to manage SSI	Nominal group technique
Number of resources used to manage SSI	Elicitation exercises
Carbon footprint for each resource	Published literature
Baseline SSI rate	GlobalSurg 2 study data[13]
Intervention effect size	Cheetah trial data[12]

© 2025 Ledda V et al. *JAMA Network Open*.

eTable 3. Parameters used in base case and sensitivity analyses

Model parameter	Best-case scenario	Base case	Worst-case scenario
Estimation of intervention carbon footprint			
Gloves carbon footprint	Lowest possible value	Mean	Highest possible value
Instrument carbon footprint	Lowest possible value	Mean	Highest possible value
Number of scrubbed staff	2 members scrubbed	3 members scrubbed	4 members scrubbed
Clinical effectiveness of intervention†	0.79 (Lower bound of 95%CI)	0.87 (absolute RR)	0.95 (Upper bound of 95% CI)
Estimation of SSI carbon footprint			
Number of wound swabs	Upper IQR (Q3)	Median	Lower IQR (Q1)
Carbon footprint of wound swabs	Highest possible value	Mean	Lowest possible value
Number of wound dressings	Upper IQR (Q3)	Median	Lower IQR (Q1)
Carbon footprint of wound dressings	Highest possible value	Mean	Lowest possible value
Number of days of antibiotics	Upper IQR (Q3)	Median	Lower IQR (Q1)
Carbon footprint of antibiotics	Highest possible value	Mean	Lowest possible value
Number of days of additional length of stay	Upper IQR (Q3)	Median	Lower IQR (Q1)
Rate of readmission*	Upper IQR (Q3)	Median	Lower IQR (Q1)
Carbon footprint of one inpatient bed day admission	Highest possible value	Mean	Lowest possible value
Rate of reattendance	Upper IQR (Q3)	Median	Lower IQR (Q1)
Carbon footprint of reattendance	Highest possible value	Mean	Lowest possible value
Rate of reoperation	Upper IQR (Q3)	Median	Lower IQR (Q1)
Carbon footprint of reoperation	Mean	80% of mean	60% of mean

* Assumed average length of stay on readmission is 3 days

† Clinical effectiveness data is based on the Cheetah trial findings and expressed as a relative risk for SSI for the intervention (glove and instrument change) versus the control

The table explains the different parameters used for the scenario modelling. Lowest possible, mean and highest possible carbon footprint values were estimated among the values found in the literature. The upper IQR, median and lower IQR numbers and rates of resource usage were estimated from the values provided by the elicitation exercises.

© 2025 Ledda V et al. *JAMA Network Open*.

eTable 4. Country of work of participants to nominal group technique meeting

Country of work	Number of participants
Benin	1
Canada	1
Ghana	2
India	2
Kenya	1
Nigeria	6
Philippines	1
Rwanda	1
United Kingdom	1
**Total**	**16**

© 2025 Ledda V et al. *JAMA Network Open*.

eTable 5. Nominal group technique meeting voting results

Should these resources be included in the carbon model to estimate the carbon footprint of a surgical site infection?	Votes (n=16)	
	Yes	No
Wound swabs	13	3
Imaging (ultrasound scan)*	10	6
Wound dressings	16	0
Antibiotics	15	1
Length of stay	16	0
Reattendance	16	0
Readmission	15	1
Reoperation	16	0

* This resource was excluded from the model as unanimous voting was not achieved and agreement was reached for its exclusion during the discussion held after the voting. Agreement was reached for the inclusion of all other resources for which voting was not unanimous.

© 2025 Ledda V et al. *JAMA Network Open*.

eTable 6. Resources suggested for inclusion in the model during nominal group technique meeting

Other resources suggested	Outcome
Type of anaesthetic used during reoperation	Already included within carbon footprint of reoperation
Packing, travel to healthcare institution for wound care	Dressing already included, travel for reattendance included in the carbon footprint extracted from literature
Pain medication	Excluded given the complexity of pain management
Patient cost deprivation, diet and loss of income	Outside the scope of the carbon model
Hospital visits for wound care	A&E reattendance already included in the model
Patient travel	Included in the carbon footprint of resources extracted from literature
Type of antibiotics used	Excluded as carbon footprint not available for all types and unlikely to significantly change results of the model

© 2025 Ledda V et al. *JAMA Network Open*.

eTable 7. Country of work of participants to elicitation exercises

Country of work	Number of participants
Austria	2
Belarus	2
Benin	3
Bosnia and Herzegovina	1
Croatia	1
Egypt	1
Ethiopia	3
France	2
Germany	1
Ghana	11
Greece	4
India	29
Italy	4
Kenya	1
Libya	1
Malaysia	6
Mexico	6
Myanmar	1
Namibia	1
Nigeria	22
Oman	1
Pakistan	2
Paraguay	1
Portugal	1
Romania	1
Rwanda	3
Serbia	1
Somalia	1
Spain	1
Sri Lanka	1
Sudan	1
Switzerland	1
Tunisia	2
United Kingdom	78
Yemen Rep.	1
**Total**	**198**

© 2025 Ledda V et al. *JAMA Network Open*.

eTable 8. Demographics of participants to elicitation exercises

Participants to elicitation exercises	N=198	%
**World Bank country income classification**		
High-income countries	97	49.0%
Low- and middle- income countries	101	51.0%
Upper middle-income countries	19	9.6%
Lower middle-income countries	73	36.9%
Low-income countries	9	4.5%
**Hospital settings**		
District/ rural hospital	43	21.7%
University (tertiary referral) hospital	155	78.3%
**Job title**		
Surgeon	162	81.8%
Nurse	3	1.5%
Missing	33	16.7%
**Surgeons' specialty**	**N=162**	**%**
Acute care surgery	1	0.6%
Breast surgery	3	1.9%
Cardiac surgery	1	0.6%
Colorectal Surgery	21	13.0%
General/ other surgery	56	34.6%
Gynaecology	4	2.5%
Hepatobiliary surgery	3	1.9%
Neurosurgery	6	3.7%
Obstetrics	3	1.9%
Oesophagogastric surgery	2	1.2%
Ophthalmology	1	0.6%
Orthopaedics	13	8.0%
Otolaryngology (head and neck surgery)	7	4.3%
Paediatric surgery	8	4.9%
Plastic surgery	9	5.5%
Surgical oncology	1	0.6%
Thoracic surgery	3	1.9%
Urology	6	3.7%
Vascular surgery	5	3.1%
Missing	9	5.5%

© 2025 Ledda V et al. *JAMA Network Open*.

eTable 9. Elicitation exercises result indicating average use of each resource in the management of an SSI

Resources	Unit	Average use (IQR)	
		LMIC	HIC
Wound swab	Number of swabs	2 (1-3)	2 (1-5)
Wound dressings	Number of dressings	9 (5-12)	7 (5-10)
Days of antibiotics	Number of days	7 (7-10)	7 (5-7)
Length of stay	Number of days	7 (5-7)	5 (3-6)
Reattendance	Rate of reattendance	6 (3-15) %	10 (5-16.5) %
Readmission*	Rate of readmission	5 (2-10) %	5 (2-10) %
Reoperation	Rate of reoperation	2 (1-5) %	2 (1-5) %

*Assumed average length of stay on readmission is 3 days

© 2025 Ledda V et al. *JAMA Network Open*.

eTable 10. Parameters for calculation of carbon footprint of glove and instrument change

Rates of gloves and instrument change	
Proportion of change of gloves in the intervention group	98.4% (6,048/6,144)
Proportion of change of gloves in the control group	0.8% (55/7,157)
Proportion of change of instruments in the intervention group	98.4% (6,047/6,144)
Proportion of change of instruments in the control group	0.2% (13/7,157)
Carbon footprint parameters	
Number of theatre staff scrubbed	3 (2-4)
Carbon footprint for gloves	0.79 (0.79-0.80) kgCO_2e
Carbon footprint for instruments*	0.33 (0.18-0.57) kgCO_2e
Total carbon footprint for the glove and instrument change	
Intervention group	2.66 (1.73-3.71) kgCO_2e
Control group	0.02 (0.01-0.03) kgCO_2e

*Needle holder, forceps, and scissors as described in the Cheetah trial
Figures in parentheses indicate range for worst- and best-case scenarios

© 2025 Ledda V et al. *JAMA Network Open*.

eTable 11. Studies used to extract the carbon footprint of resources for estimating the SSI of glove and instrument changes, as well as the SSI carbon footprint

Resource	Studies	Country	Functional unit in the present study	Methodological approach and study boundaries	Notes	Estimated footprint (KgCO_2e)
**Surgical gloves**	Jamal et al.[14]	United Kingdom	One pair of gloves	LCA, cradle to grave	Latex	0.79
					Non-latex	0.80
					**Mean**	**0.79**
**Reusable surgical instrument**	Rizan C et al.[15]	United Kingdom	One individual instrument	Process-based carbon footprint, post-use to pre-use	Wrapped in pouch	0.19
					In aluminium container	0.07
					In tray	0.06
					**Mean**	**0.11**
**Wound swab**	Ling et al.[16]	China	One individual swab	LCA, cradle to grave	As part of COVID-19 nucleic acid test	**0.61**
**Wound dressing**	Rizan C at al.[17]	United Kingdom	One individual dressing	Carbon footprint, cradle to grave	Non-woven dressing – carpal tunnel decompression	0.01
					Non-woven 10x20cm – hernia repair	0.02
					Non-woven 10x30cm – knee arthroplasty	0.05
					Border dressing 10x30 cm- knee arthroplasty	0.06
					Border dressing 6x8cm – knee arthroplasty	0.01
					Non-woven dressing 6x7cm – laparoscopic cholecystectomy	0.03
					**Mean**	**0.03**
**Antibiotics**	Brunet et al.[18]	Spain	One day of antibiotics (penicillin V)	LCA, cradle to manufacturer gate	Assuming dose of 500 mg four times a day	0.01
	Weisz et al.[19]	Austria	One day of antibiotics (Amoxicillin)	LCA, cradle to manufacturer gate	Assuming a dose of 500 mg three times a day	0.02
					**Mean**	**0.02**
	Thiel et al.[20]	USA	One clinic visit per patient	LCA, cradle to grave	Acute care surgery	23.19

© 2025 Ledda V et al. *JAMA Network Open*.

**Reattendance**	Kponee-Shovein et al.[21]	United Kingdom	One visit for patient	Carbon footprint, pharmaceuticals and medical services used during acute exacerbations	Acute asthma exacerbation	67.25
	Connor et al.[22]	United Kingdom	Outpatient appointment	Component analysis, cradle to grave	Renal services	22
					**Mean**	**37.48**
**Readmission/ length of stay**	Zhang et al. [23]	Germany	One bed day	Environmentally-extended input-output analysis, admission to discharge	Acute decompensated heart failure patient	32
	Tennison et al.[24]	United Kingdom	One bed day	Hybrid carbon footprint, direct and indirect impacts	-	125
	Connor et al.[22]	United Kingdom	One bed day	Component analysis, cradle to grave	Renal services	161
					**Mean**	**106**
**Reoperation**	MacNeill et al.[25]	United Kingdom/ Canada/USA	Per procedure	Carbon footprint. Operating room door-to-door (intra-operative period)	Vancouver General Hospital	146
					University of Minnesota Medical Centre	232
					John Radcliffe Hospital	173
					**Mean**	**183.7**

LCA: life-cycle assessment.

© 2025 Ledda V et al. *JAMA Network Open*.

eTable 12. Country-specific SSI rates for clean-contaminated and contaminated-dirty procedures extracted from GlobalSurg study

		SSI rate	
**Country**	**Country income group**	**Clean-contaminated procedures**	**Contaminated-dirty procedures**
Andorra	HIC	9.18%	17.89%
Antigua and Barbuda	HIC	8.74%	17.10%
Australia	HIC	10.40%	20.00%
Austria	HIC	9.55%	18.55%
Bahamas	HIC	8.76%	17.13%
Bahrain	HIC	8.90%	17.39%
Barbados	HIC	8.75%	17.11%
Belgium	HIC	9.63%	18.68%
Brunei Darussalam	HIC	9.27%	18.06%
Canada	HIC	10.11%	19.49%
Chile	HIC	8.96%	17.51%
Croatia	HIC	8.86%	17.32%
Cyprus	HIC	9.24%	18.00%
Czech Republic	HIC	9.37%	18.23%
Denmark	HIC	10.32%	19.85%
Estonia	HIC	9.31%	18.11%
Finland	HIC	9.52%	18.48%
France	HIC	9.59%	18.61%
Germany	HIC	10.23%	19.71%
Greece	HIC	9.34%	18.17%
Guyana	HIC	14.09%	26.09%
Hong Kong, China (SAR)	HIC	9.98%	19.29%
Hungary	HIC	8.92%	17.43%
Iceland	HIC	9.82%	19.01%
Ireland	HIC	10.19%	19.64%
Israel	HIC	9.74%	18.88%
Italy	HIC	9.41%	18.29%
Japan	HIC	9.67%	18.74%
Korea (Republic of)	HIC	9.78%	18.94%
Kuwait	HIC	8.84%	17.29%
Latvia	HIC	8.88%	17.35%
Liechtenstein	HIC	9.94%	19.22%
Lithuania	HIC	9.09%	17.73%
Luxembourg	HIC	9.71%	18.81%
Malta	HIC	9.06%	17.68%
Netherlands	HIC	10.27%	19.78%

© 2025 Ledda V et al. *JAMA Network Open*.

New Zealand	HIC	10.07%	19.42%
Norway	HIC	10.45%	20.07%
Oman	HIC	8.79%	17.19%
Panama	HIC	8.74%	17.09%
Poland	HIC	9.12%	17.78%
Portugal	HIC	8.94%	17.46%
Qatar	HIC	9.21%	17.94%
Romania	HIC	8.78%	17.17%
Saint Kitts and Nevis	HIC	8.97%	17.51%
Saudi Arabia	HIC	9.04%	17.64%
Seychelles	HIC	8.75%	17.11%
Singapore	HIC	10.02%	19.35%
Slovakia	HIC	9.15%	17.84%
Slovenia	HIC	9.48%	18.42%
Spain	HIC	9.44%	18.36%
Sweden	HIC	9.90%	19.15%
Switzerland	HIC	10.36%	19.92%
Trinidad and Tobago	HIC	8.75%	17.12%
United Arab Emirates	HIC	8.99%	17.55%
United Kingdom	HIC	9.86%	19.08%
United States	HIC	10.15%	19.57%
Uruguay	HIC	8.77%	17.15%
Afghanistan	LIC	30.74%	47.82%
Burkina Faso	LIC	36.54%	53.79%
Burundi	LIC	37.03%	54.27%
Central African Republic	LIC	37.03%	54.27%
Chad	LIC	37.03%	54.27%
Congo (Democratic Republic of the)	LIC	33.13%	50.35%
Eritrea	LIC	37.03%	54.27%
Ethiopia	LIC	32.17%	49.34%
Gambia	LIC	32.64%	49.85%
Guinea-Bissau	LIC	34.09%	51.34%
Liberia	LIC	33.61%	50.85%
Madagascar	LIC	23.26%	39.07%
Malawi	LIC	31.69%	48.84%
Mali	LIC	34.58%	51.84%
Mozambique	LIC	35.07%	52.33%
Rwanda	LIC	27.52%	44.22%
Sierra Leone	LIC	35.56%	52.82%
South Sudan	LIC	29.81%	46.80%
Sudan	LIC	28.88%	45.77%

© 2025 Ledda V et al. *JAMA Network Open*.

Syrian Arab Republic	LIC	16.52%	29.81%
Togo	LIC	26.63%	43.18%
Uganda	LIC	27.97%	44.73%
Yemen	LIC	25.76%	42.15%
Algeria	LMIC	9.20%	17.90%
Angola	LMIC	21.34%	36.58%
Bangladesh	LMIC	18.91%	33.27%
Benin	LMIC	28.42%	45.25%
Bhutan	LMIC	15.98%	29.01%
Bolivia (Plurinational State of)	LMIC	13.09%	24.50%
Cambodia	LMIC	19.24%	33.73%
Cameroon	LMIC	22.87%	38.57%
Cape Verde	LMIC	13.67%	25.43%
Comoros	LMIC	25.33%	41.63%
Congo	LMIC	17.08%	30.64%
Cote d'Ivoire	LMIC	31.21%	48.33%
Djibouti	LMIC	29.34%	46.28%
Egypt	LMIC	11.34%	21.60%
Ghana	LMIC	18.27%	32.37%
Guinea	LMIC	36.05%	53.30%
Haiti	LMIC	27.07%	43.70%
Honduras	LMIC	15.72%	28.62%
India	LMIC	15.47%	28.24%
Iran (Islamic Republic of)	LMIC	8.81%	17.22%
Jordan	LMIC	9.08%	17.69%
Kenya	LMIC	19.91%	34.66%
Kiribati	LMIC	17.36%	31.06%
Kyrgyzstan	LMIC	13.28%	24.80%
Lao People's Democratic Republic	LMIC	18.59%	32.82%
Lebanon	LMIC	8.78%	17.17%
Lesotho	LMIC	26.19%	42.66%
Mauritania	LMIC	24.08%	40.09%
Micronesia (Federated States of)	LMIC	13.88%	25.76%
Mongolia	LMIC	9.72%	18.81%
Morocco	LMIC	14.53%	26.78%
Myanmar	LMIC	20.97%	36.10%
Nepal	LMIC	20.26%	35.13%
Nicaragua	LMIC	14.30%	26.43%
Nigeria	LMIC	22.48%	38.07%
Pakistan	LMIC	20.61%	35.61%
Papua New Guinea	LMIC	24.91%	41.12%
Philippines	LMIC	12.39%	23.35%

© 2025 Ledda V et al. *JAMA Network Open*.

Samoa	LMIC	10.96%	20.96%
Sao Tome and Principe	LMIC	19.57%	34.19%
Senegal	LMIC	30.27%	47.31%
Solomon Islands	LMIC	24.49%	40.60%
Sri Lanka	LMIC	8.87%	17.32%
Swaziland	LMIC	21.71%	37.07%
Tajikistan	LMIC	15.23%	27.86%
Tanzania (United Republic of)	LMIC	22.09%	37.57%
Timor-Leste	LMIC	16.25%	29.41%
Tunisia	LMIC	10.03%	19.36%
Ukraine	LMIC	9.16%	17.83%
Uzbekistan	LMIC	12.22%	23.08%
Vanuatu	LMIC	16.79%	30.22%
Vietnam	LMIC	12.91%	24.20%
Zambia	LMIC	17.96%	31.93%
Zimbabwe	LMIC	23.67%	39.58%
Albania	UMIC	9.29%	18.07%
Argentina	UMIC	9.01%	17.59%
Armenia	UMIC	9.35%	18.16%
Azerbaijan	UMIC	9.01%	17.56%
Belarus	UMIC	8.81%	17.23%
Belize	UMIC	10.51%	20.19%
Bosnia and Herzegovina	UMIC	9.40%	18.26%
Botswana	UMIC	11.08%	21.17%
Brazil	UMIC	8.92%	17.41%
Bulgaria	UMIC	8.74%	17.10%
China	UMIC	9.58%	18.57%
Colombia	UMIC	10.12%	19.52%
Costa Rica	UMIC	8.80%	17.19%
Cuba	UMIC	8.77%	17.15%
Dominica	UMIC	9.87%	19.08%
Dominican Republic	UMIC	10.62%	20.37%
Ecuador	UMIC	9.46%	18.36%
El Salvador	UMIC	12.56%	23.62%
Equatorial Guinea	UMIC	17.66%	31.49%
Fiji	UMIC	9.65%	18.69%
Gabon	UMIC	11.62%	22.07%
Georgia	UMIC	8.95%	17.45%
Grenada	UMIC	9.04%	17.62%
Guatemala	UMIC	14.99%	27.50%
Indonesia	UMIC	11.76%	22.31%
Iraq	UMIC	13.47%	25.11%

© 2025 Ledda V et al. *JAMA Network Open*.

Jamaica	UMIC	10.31%	19.84%
Kazakhstan	UMIC	8.75%	17.12%
Libya	UMIC	9.95%	19.22%
Macedonia	UMIC	9.11%	17.75%
Malaysia	UMIC	8.74%	17.09%
Maldives	UMIC	10.84%	20.76%
Mauritius	UMIC	8.74%	17.10%
Mexico	UMIC	8.89%	17.36%
Moldova (Republic of)	UMIC	11.21%	21.38%
Montenegro	UMIC	8.83%	17.26%
Namibia	UMIC	14.75%	27.13%
Palau	UMIC	8.74%	17.09%
Palestine, State of	UMIC	12.07%	22.81%
Paraguay	UMIC	11.91%	22.56%
Peru	UMIC	9.25%	17.98%
Russian federation	UMIC	8.80%	17.21%
Saint Lucia	UMIC	9.52%	18.46%
Saint Vincent and the Grenadines	UMIC	10.21%	19.67%
Serbia	UMIC	8.76%	17.13%
South Africa	UMIC	12.73%	23.91%
Suriname	UMIC	10.73%	20.56%
Thailand	UMIC	9.79%	18.94%
Tonga	UMIC	10.41%	20.01%
Turkey	UMIC	8.85%	17.28%
Turkmenistan	UMIC	11.48%	21.83%
Venezuela (Bolivarian Republic of)	UMIC	8.83%	17.25%

© 2025 Ledda V et al. *JAMA Network Open*.

eFigure 1. Flowchart of votes from elicitation exercises included in the study

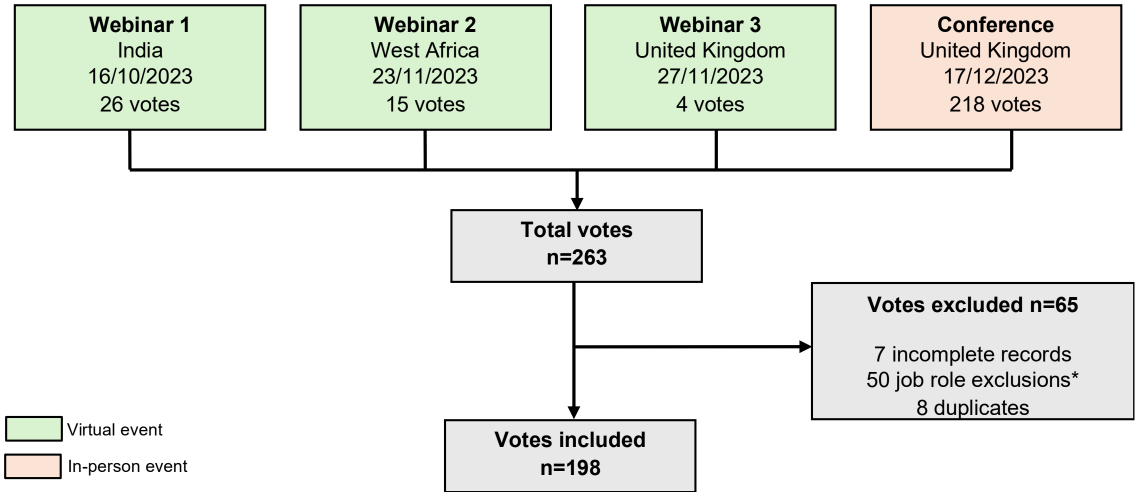

*Job roles excluded included public health doctors or other specialty doctors, research members, theatre staff, members of hospital management and industry members who are not regularly involved in the management of surgical wounds.

© 2025 Ledda V et al. *JAMA Network Open*.

eReferences

1. Drew J, Christie SD, Rainham D, Rizan C. HealthcareLCA: an open-access living database of health-care environmental impact assessments. *The Lancet Planetary Health* 2022; **6**(12): e1000-e12.
2. Edwards M, Graziadio S, Shore J, et al. Plus Sutures for preventing surgical site infection: a systematic review of clinical outcomes with economic and environmental models. *BMC Surgery* 2023; **23**(1): 300.
3. Gumera A, Mil M, Hains L, Fanshaw SR, Dunne B. Reusable surgical headwear has a reduced carbon footprint and matches disposables regarding surgical site infection: a systematic review and meta-analysis. *Journal of Hospital Infection* 2024; **152**: 164-72.
4. Parker EB, Bluman EM, Chiodo CP, Martin EA, Smith JT. Carbon Footprint of Minor Foot and Ankle Surgery: A Randomized Controlled Trial. *Foot Ankle Orthop* 2024; **9**(1): 24730114241238231.
5. Friedericy HJ, Friedericy AF, de Weger A, et al. Effect of unidirectional airflow ventilation on surgical site infection in cardiac surgery: environmental impact as a factor in the choice for turbulent mixed air flow. *Journal of Hospital Infection* 2024; **148**: 51-7.
6. Van de Ven AH, Delbecq AL. The Nominal Group as a Reseach Instrument for Exploratory health studies. *American Journal of Public Health* 1972; **March**: 337-42.
7. Harvey N, Holmes CA. Nominal group technique: An effective method for obtaining group consensus. *International Journal of Nursing Practice* 2012; **18**(2): 188-94.
8. Rubin G, Wit ND, Meineche-Schmidt V, Seifert B, Hall N, Hungin P. The diagnosis of IBS in primary care: consensus development using nominal group technique. *Family Practice* 2006; **23**(6): 687-92.
9. Spurlock DR, Cooper S, Cant R, et al. The Nominal Group Technique: Generating Consensus in Nursing Research. *Journal of Nursing Education* 2020; **59**(2): 65-7.
10. Ademuyiwa A, Picciochi M. Conference report: NIHR Global Surgery Unit: Lagos, Nigeria 2023. BJS Academy: BJS, 2023.
11. Ledda V. Conference report: Research for Greener Surgery 2023 at the University of Birmingham. BJS Academy: BJS, 2024.
12. Andoh AB, Atindaana Francis A, Abdulkarim AA, et al. Routine sterile glove and instrument change at the time of abdominal wound closure to prevent surgical site infection (ChEETAh): a pragmatic, cluster-randomised trial in seven low-income and middle-income countries. *The Lancet* 2022; **400**(10365): 1767-76.
13. Bhangu A, Ademuyiwa AO, Aguilera ML, et al. Surgical site infection after gastrointestinal surgery in high-income, middle-income, and low-income countries: a prospective, international, multicentre cohort study. *The Lancet Infectious Diseases* 2018; **18**(5): 516-25.
14. Jamal H, Lyne A, Ashley P, Duane B. Non-sterile examination gloves and sterile surgical gloves: which are more sustainable? *Journal of Hospital Infection* 2021; **118**: 87-95.
15. Rizan C, Lillywhite R, Reed M, Bhutta MF. Minimising carbon and financial costs of steam sterilisation and packaging of reusable surgical instruments. *British Journal of Surgery* 2021; **109**(2): 200-10.
16. Ji L, Wang Y, Xie Y, et al. Potential Life-Cycle Environmental Impacts of the COVID-19 Nucleic Acid Test. *Environ Sci Technol* 2022; **56**(18): 13398-407.
17. Rizan C, Lillywhite R, Reed M, Bhutta MF. The carbon footprint of products used in five common surgical operations: identifying contributing products and processes. *J R Soc Med* 2023; **116**(6): 199-213.
18. Brunet R, Guillén-Gosálbez G, Jiménez L. Combined simulation–optimization methodology to reduce the environmental impact of pharmaceutical processes: application to the production of Penicillin V. *Journal of Cleaner Production* 2014; **76**: 55-63.
19. Weisz U, Pichler P-P, Jaccard IS, et al. Carbon emission trends and sustainability options in Austrian health care. *Resources, Conservation and Recycling* 2020; **160**: 104862.

© 2025 Ledda V et al. *JAMA Network Open*.

20. Thiel CL, Mehta N, Sejo CS, et al. Telemedicine and the environment: life cycle environmental emissions from in-person and virtual clinic visits. *npj Digital Medicine* 2023; **6**(1): 87.
21. Kponee-Shovein K, Marvel J, Ishikawa R, et al. Carbon footprint and associated costs of asthma exacerbation care among UK adults. *J Med Econ* 2022; **25**(1): 524-31.
22. Connor A, Lillywhite R, Cooke MW. The carbon footprint of a renal service in the United Kingdom. *QJM: An International Journal of Medicine* 2010; **103**(12): 965-75.
23. Zhang X, Albrecht K, Herget-Rosenthal S, Rogowski WH. Carbon footprinting for hospital care pathways based on routine diagnosis-related group (DRG) accounting data in Germany: An application to acute decompensated heart failure. *Journal of Industrial Ecology* 2022; **26**(4): 1528-42.
24. Tennison I, Roschnik S, Ashby B, et al. Health care's response to climate change: a carbon footprint assessment of the NHS in England. *The Lancet Planetary Health* 2021; **5**(2): e84-e92.
25. MacNeill AJ, Lillywhite R, Brown CJ. The impact of surgery on global climate: a carbon footprinting study of operating theatres in three health systems. *The Lancet Planetary Health* 2017; **1**(9): e381-e8.

© 2025 Ledda V et al. *JAMA Network Open*.
