# Supplementary material for: Modeled Carbon Footprint of Change of Sterile Gloves and Instruments for Abdominal Wound Closure
Source: JAMA Netw Open. 2025 Aug 6;8(8):e2525355. doi: 10.1001/jamanetworkopen.2025.25355 (PMC12329605; doi:10.1001/jamanetworkopen.2025.25355)
Supplement: Supplement 2. — Nonauthor Collaborators [file jamanetwopen-e2525355-s002.pdf]

Supplemental Online Content: Nonauthor Collaborators
*First name, last name, and suffix (if applicable) are required and will appear in PubMed.

*Group Name(s): National Institute for Health and Care Research Global Health Research Unit on Global Surgery							
***First Name and Middle Initial(s)**	***Last Name**	***Suffix (eg, Jr, III)**	Academic Degrees	Institution	Location (city, state/province, country)	Role or Contribution, eg, chair, principal investigator	Group (if more than 1 Group listed in the byline) and/or Subgroup (eg, Steering Committee)
Abbey-Louise	Matthews	N/A					
Ahmed	Abdisamed	N/A					
Adamu	Issaka	N/A					
Ahmad Hassan	Jibril	N/A					
AHMED ALBAGIR	ALI ALTAYYEB	N/A					
Ahmed	Mekki	N/A					
Aidan	Bannon	N/A					
Aikaterini	Karakonstanti	N/A					
Aime	Hirwa						
Albaro Jose	Nieto-Calvache						
Alejandro	Gonzalez-Ojeda						
Alfie J	Kavalakat						
Amar	Odedra						
Amman	Malik						
Andrea	Nickeas						
Andrew	Stevenson						
Andrey	Litvin						
Angelika	Kaufmann						
Anil	Luther						
Anis	Hasnaoui						
Anisa	Kushairi						
Anja	Imsirovic						
Anne	Robinson						
Antonio	Perez-Ferrer						
Antonio Ramos	De-la-Medina						
Anu Susan	George						
April C	Roslani						
Aristeidis	Papadopoulos						
Arun	Sahni						
Ashish	Chaudhrie						

Supplemental Online Content: Nonauthor Collaborators
*First name, last name, and suffix (if applicable) are required and will appear in PubMed.

***First Name and Middle Initial(s)**	***Last Name**	***Suffix (eg, Jr, III)**	Academic Degrees	Institution	Location (city, state/province, country)	Role or Contribution, eg, chair, principal investigator	Group (if more than 1 Group listed in the byline) and/or Subgroup (eg, Steering Committee)
Ashish	Tirkey						
Ashly	Thomas						
Ayesha	Bibi						
Bashir abobaker	albakosh						
Binay	Kumar						
Branko	Bogdanic						
Bruno	Nardo						
Bryony	David						
Caitlin	Brennan						
Cara	Hatcher						
Carolina Moreno	Licea						
Caroline	Wilburn						
Catriona	Frankling						
CHAMAIDI	SARAKATSIANO U						
Chinar	Goyal						
CHARITAKI	EVGENIA						
Chris J	Smart						
Christian	Agbo						
Christian	Udu Ngwu						
Christiana	Osei-Dwomoh						
Christopher	Aboah						
Claudia	Castellanos						
Cleo	Kenington						
CLOTILDE	FUENTES OROZCO						
Cortland	Linder						
CYNTHIA	AYODEJI AGBONROFO						
Deena	Harji						
Deepak	Jain						
Deepak	Singh						
Dimitrios	Spinos						
Djifid	Morel Seto						

Supplemental Online Content: Nonauthor Collaborators
*First name, last name, and suffix (if applicable) are required and will appear in PubMed.

*First Name and Middle Initial(s)	*Last Name	*Suffix (eg, Jr, III)	Academic Degrees	Institution	Location (city, state/province, country)	Role or Contribution, eg, chair, principal investigator	Group (if more than 1 Group listed in the byline) and/or Subgroup (eg, Steering Committee)
Dmitry	Adamovich						
Dorothy	Kufeji						
Doug	Bowley						
Abubakar	Bala Muhammad						
Gareth	Thompson						
Narendra	Siddaiah						
Subham	Jakhar						
Dragana	Zivkovic						
Ebenezer	Kwame Amofa						
Ebere	Osinachi Ugwu						
Eleanor	Cotton						
Elisa	Paoluzzi Tomada						
Elizabeth	Li						
Elizabeth	Westwood						
Ella	Wheeley						
Emmanuel A.	Nachelleh						
Emmet	Dorrian						
Eseenam	Agbeko						
Ewen	Harrison						
fahed	gareb						
Fareeda	Galley						
Fennie	Sam						
Feriha Fatima	Khidri						
Francesco	Pata						
Gianluca	Pellino						
Gonzalo	Delgado-Hernández						
GUILLERMO	YANOWSKY REYES						
Gustavo Miguel	Machain						
Habeeb	Hanafi						
Hadijat	Olaide Raji						
HANA	SIDDIG HAMMAD HASSAN						
Haris	Kuralić						

Supplemental Online Content: Nonauthor Collaborators
*First name, last name, and suffix (if applicable) are required and will appear in PubMed.

*First Name and Middle Initial(s)	*Last Name	*Suffix (eg, Jr, III)	Academic Degrees	Institution	Location (city, state/province, country)	Role or Contribution, eg, chair, principal investigator	Group (if more than 1 Group listed in the byline) and/or Subgroup (eg, Steering Committee)
Harry	wilson						
Helen	van Vliet						
Helen	Suttenwood						
Hesham	Abozied						
Hesham	Zalghana						
hossam	Eslam Mohammed salah						
Hugh	Montgomery						
Humaira	Hussain						
Ibrahim Adel	Hamdoun						
Ifeanyichukwu	Chinedum Ugwu						
Imtiaz	Wani						
Iniesta	aurelie						
Isam	Bsisu						
Ismail	LAWANI						
Isobel	seddon						
JAMEEL	ISMAIL AHMAD						
James	Glasbey						
Jane	Barnard						
Jayan	Dewantha Jayasinghe						
Jennifer	Kirkby						
Jennifer	Ip						
Jessica	Fleminger						
Joël L.	Lavanchy						
John	Tabiri Abebrese						
Jon	LACY-COLSON						
Jonathan	Lee						
Jonathan P	Evans						
Kai Hui	Loo						
Katherina	McEvoy						
Kethy	FAGNON						
Khaled Mahmoud A	Omar						
Khaled Mohammed	Al-Sayaghi						

Supplemental Online Content: Nonauthor Collaborators
*First name, last name, and suffix (if applicable) are required and will appear in PubMed.

*First Name and Middle Initial(s)	*Last Name	*Suffix (eg, Jr, III)	Academic Degrees	Institution	Location (city, state/province, country)	Role or Contribution, eg, chair, principal investigator	Group (if more than 1 Group listed in the byline) and/or Subgroup (eg, Steering Committee)
Konstancja	Tadrak						
Kriscia Vanessa	Ascencio Diaz						
Lanre	Lamid						
Laura	Ballance						
Lofty-John	Chukwuemeka Anyanwu						
Lovenish	Bains						
Ludger	Barthelmes						
Luke	Nicholson						
Madushika	Rajapakse						
Margot	Flint						
Mario Jesús	Guzmán Ruvalcaba						
Mark	Cheetham						
Marta	Wachtl						
Massimiliano	Veroux						
Matthew	Gardiner						
Matthew	Popplewell						
Michelle	Spiteri						
Miguel	GASAKURE						
Minale	Merene						
moath Ahmed	Abdullah almuradi						
Mohamed	Thaha						
Mohamed	Ghula						
Mohammad	Marar						
Mohammed	Sheriff						
Mohammed	Salele Aliyu						
Montassar	Ghalleb						
Moses	Dokurugu						
Muhammad Fairuz	Shah Abd Karim						
muhammad farzree	bin mohd ismail						
Muhammad	Mudasir Khan						
Navneet	Kumar Chaudhary						
Nick	Battersby						

Supplemental Online Content: Nonauthor Collaborators
*First name, last name, and suffix (if applicable) are required and will appear in PubMed.

***First Name and Middle Initial(s)**	***Last Name**	***Suffix (eg, Jr, III)**	Academic Degrees	Institution	Location (city, state/province, country)	Role or Contribution, eg, chair, principal investigator	Group (if more than 1 Group listed in the byline) and/or Subgroup (eg, Steering Committee)
Nida	Wahid Bashir						
Nnaemeka	Nwafulume						
Omolara	Williams						
panna	patel						
Pariza	Gupta						
Patrick	Sharman						
Paul	Marriott						
Paul	Robinson						
PETER	IKPONMWOSA AGBONROFO						
Peter	Paal						
Rachel	Sam						
Rahel	Abebayehu Assefa						
Raja	Haseeb Basit						
Rajive	Jose						
Rajkumar	KS						
RAM	PRASAD SUBEDI						
Ramanpreet	Kaur						
rasiah	bharathan						
Rawoof	Mohammed						
Reddy	Abhinaya P						
Robert	Parker						
Robert	Whitham						
Rohin	Mittal						
romain	Letartre						
Romy	Kenyon						
Rory	F Kokelaar						
Ross	Lathan						
Ross	Coomber						
Ruzaimie	Noor						
Salih	Al-Ani						
Saminu	Muhammad						
Samson	Olori						

*First name, last name, and suffix (if applicable) are required and will appear in PubMed.

***First Name and Middle Initial(s)**	***Last Name**	***Suffix (eg, Jr, III)**	Academic Degrees	Institution	Location (city, state/province, country)	Role or Contribution, eg, chair, principal investigator	Group (if more than 1 Group listed in the byline) and/or Subgroup (eg, Steering Committee)
Samuel	Ali Sani						
Samuel	Kwame Amoako Asirifi						
sanjay	pandanaboyana						
Sankar	Balakrishnan						
Setthasorn	Ooi						
Shireen Anne	Nah						
Shivani	Aggarwal						
simon	Clarke						
Sonia	Bhangu						
Sonia	Mathai						
Soyombo	Orsoo						
Spiros	Delis						
Stefan	Welter						
Stelian Stefanita	Mogoanta						
Stephen	Gboya Gana						
Suraiya	Auwal Suleiman						
Taiye	Taibat Ibiyeye						
Tariq	Alhammali						
Theophilus	Anyomih						
Theophilus Justus	Kofi Adjeso						
Thida	Oung						
Tom	Challoner						
Tosin	Olusoga Akinyemi						
Upamanyu	Nath						
Uzair	Khan						
V	Pollet						
Vairavan	Narayanan						
Vandana	chaukar						
Vasanthika	Thuduvage						
W M C	Alwis						
Wegene	Tadesse Shenkutie						
Yousuf	Sabah						

Supplemental Online Content: Nonauthor Collaborators
*First name, last name, and suffix (if applicable) are required and will appear in PubMed.

***First Name and Middle Initial(s)**	***Last Name**	***Suffix (eg, Jr, III)**	Academic Degrees	Institution	Location (city, state/province, country)	Role or Contribution, eg, chair, principal investigator	Group (if more than 1 Group listed in the byline) and/or Subgroup (eg, Steering Committee)
Zahra	Hussain						
