# Supplementary material for: Modeled Carbon Footprint of Change of Sterile Gloves and Instruments for Abdominal Wound Closure
Source: JAMA Netw Open. 2025 Aug 6;8(8):e2525355. doi: 10.1001/jamanetworkopen.2025.25355 (PMC12329605; doi:10.1001/jamanetworkopen.2025.25355)
Supplement: Supplement 3. — Data Sharing Statement [file jamanetwopen-e2525355-s003.pdf]

Data Sharing Statement

Data
Data available: No

Additional Information
Explanation for why data not available: No new dataset has been created
